# Microstructure and Texture Evolution of AZ31 Alloy Prepared by Cyclic Expansion Extrusion with Asymmetrical Extrusion Cavity at Different Temperatures

**DOI:** 10.3390/ma13173757

**Published:** 2020-08-25

**Authors:** Jie Zheng, Zhaoming Yan, Qiang Wang, Zhimin Zhang, Yong Xue

**Affiliations:** School of Materials Science and Engineering, North University of China, Taiyuan 030051, China; cqzhengjie@163.com (J.Z.); zmyan1027@126.com (Z.Y.); npu_ydwang@163.com (Q.W.); forge_zmzhang@126.com (Z.Z.)

**Keywords:** magnesium alloy, microstructure, texture evolution, cyclic expansion-extrusion

## Abstract

This work is to study the microstructure and texture evolution of AZ31 alloy prepared by cyclic expansion extrusion with an asymmetrical extrusion cavity (CEE-AEC) at different deformation temperatures. The result shows AZ31 alloy undergoes continuous dynamic recrystallization (CDRX) and discontinuous dynamic recrystallization (DDRX) during CEE-AEC processing. At the initial stage of deformation, AZ31 alloys exhibit similar bimodal microstructure of coarse deformed grains surrounded by fine DRXed grains. As the passes increase, the cumulative strain increases, and the coarse grains of all samples are almost replaced by fine equiaxed grains. The average grain sizes and the basal texture intensities of the deformed samples increase as the deformation temperature increases. In addition, due to the existence of an asymmetrical cavity, as the passes increase, the basal textures of all samples are deflected with maximum intensities increase, and even an unusual bimodal texture is formed, resulting in a soft orientation that is easy to basal slip.

## 1. Introduction

In recent years, magnesium (Mg) and its alloys have received attention from the automotive industry and the aerospace field due to their high specific stiffness and excellent lightweight performance [1,2,3]. However, Mg alloys have poor formability and ductility at room temperature, which limits their wide application. This is mainly attributed to the insufficient independent sliding systems of Mg alloys due to the hexagonal close-packed (hcp) crystal structure [4,5]. Therefore, many researchers are committed to proposing different technologies to improve the mechanical properties of Mg alloys [6,7,8].

At present, grain refinement and texture control are the most effective methods for preparing high-performance Mg alloys [9,10,11]. Severe plastic deformation techniques, such as equal-channel angular pressing (ECAP) [12,13], high pressure torsion (HPT) [14,15], cyclic expansion-extrusion (CEE) [16,17], and high-pressure and high-temperature (HPHT) [18,19] significantly refine grains by increasing the cumulative strain during deformation. Matsushita et al. [20] prepared high-performance Mg85Y9Zn6 (wt. %) alloy with a compression yield strength of 780MPa through HPHT technology. Yan et al. [21] prepared the Mg97Y2Zn1 (wt. %) alloy wire with novel band microstructure by combined ECAP and heavy drawing; the ultimate yield strength (UTS) and elongation-to-failure are 570 MPa and 12%, respectively. In addition, some studies have shown that the asymmetrical cavity can introduce shear deformation during the deformation process to control the texture evolution and grain refinement. Wang et al. [22] introduced shear deformation into the traditional extruded sheet process by changing the cavity structure to form deflection basal texture, which effectively improved the mechanical properties of AZ31 alloy sheets. Song et al. [23] effectively weakened the basal texture of AZ31 sheets by introducing a continuous bending channel into equal channel angular rolling. Wang et al. [24] showed that a staggered extrusion process can significantly refine the grains compared with traditional extrusion.

Based on the above research progress, it is shown that the shear deformation introduced by changing the cavity structure can significantly refine the grains and improve the mechanical properties. Therefore, we have proposed a new severe plastic deformation technology, which is called cyclic expansion extrusion with an asymmetrical extrusion cavity (CEE-AEC). By introducing an asymmetric cavity in the die, the shear deformation is combined with the upsetting and extrusion deformation in a single pass.

Further, we studied the microstructure and mechanical properties of Mg-Gd-Y-Zn-Zr alloy prepared by the CEE-AEC process [25,26]. The alloy contains many long-period stacking ordered (LPSO) phases, which significantly affect the microstructure and texture evolution during deformation. The resulting effect of the CEE-AEC process on the microstructure and texture evolution is still unclear. Therefore, in this paper, we investigated the effect of different temperatures on the microstructure, texture evolution, and DRX mechanism of AZ31 alloy during the CEE-AEC deformation process.

## 2. Materials and Methods

**Figure 1:**
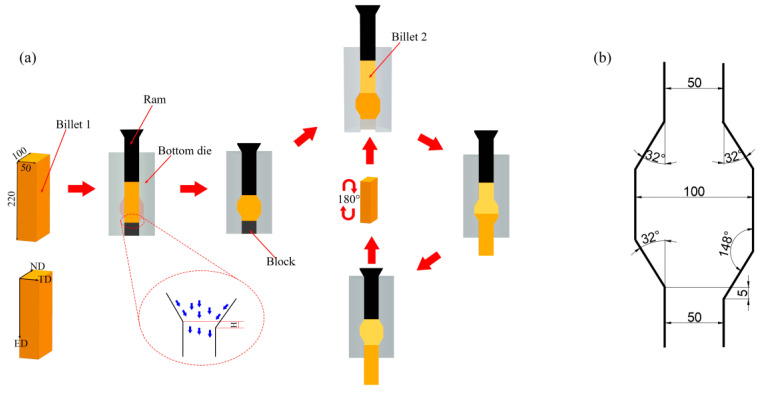
(**a**) The schematic of the cyclic expansion extrusion with an asymmetrical extrusion cavity (CEE-AEC) process; (**b**) the specific parameters of the mold cavity.

As-cast commercial AZ31 Mg alloy (Mg-2.96Al-0.89Zn, wt. %) with a length of 300 mm and a diameter of 220 mm was produced by semi-continuous casting by Shanxi Wenxi Yinguang Magnesium Industry Group (Shanxi Province, China). Billets with a width of 50 mm, a length of 100 mm, and a height of 220 mm were obtained by machining and then homogenizing in an electronic furnace (North University of China, Taiyuan, Shanxi Province, China) at 400 °C for 12 h. Then, the homogenized billets were extruded using CEE-AEC dies with different temperatures. The schematic diagram of the CEE-AEC process and specific parameters of the cavity are shown in Figure 1. Before the deformation, the homogenized billets and the CEE-AEC dies were reheated to the corresponding deformation temperature and retained for 30 min. The three-pass CEE-AEC experiments were performed at different temperatures. The load of the hydraulic press (North University of China, Taiyuan, Shanxi Province, China) was 630 KN, and the extrusion speed was 1 mm/s. Oil-based graphite (North University of China, Taiyuan, Shanxi Province, China) was used as a lubricant during extrusion.

**Figure 2:**
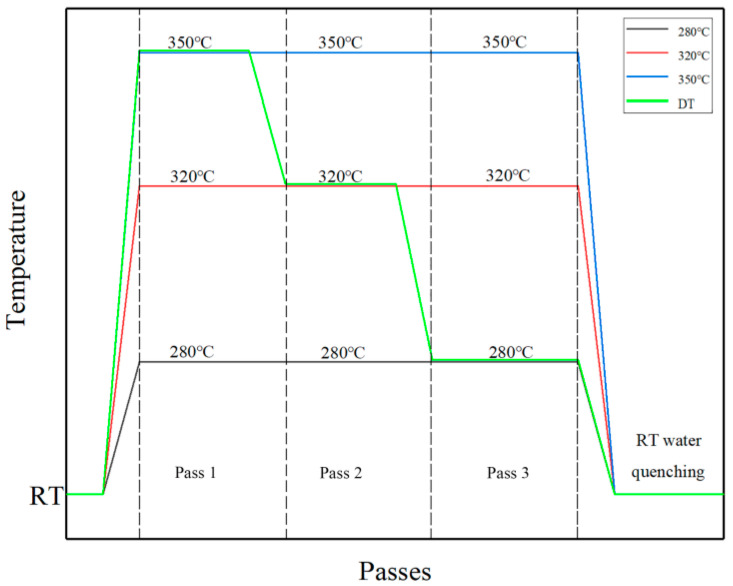
The schematic diagram of the experiment procedure.

Figure 2 reveals the schematic diagram of the CEE-AEC experiment procedure. The experiments were divided into four groups: the first group was isothermal deformation, and the deformation temperature was 280 °C; the second group was isothermal deformation, and the deformation temperature was 320 °C; the third group was isothermal deformation, and the deformation temperature was 350 °C; the fourth group temperature was decreased from 350 °C to 280 °C pass by pass. The first drop was 30 °C, and the second drop was 40 °C. The decreased temperature of the fourth group sample was defined as the DT sample. After each group of experiments completed the final pass, the samples were quenched in water (North University of China, Taiyuan, China) at 25 °C. A description for three orthogonal directions of the deformed billets in this paper is ED for the extrusion direction (height), TD for the transverse direction (width), and ND for the normal direction (length). Samples for microstructure observation and texture analysis were machined from the as-cast and deformed billets, and the observation plane was the ED × TD plane parallel to the ED. Before optical microscope (OM, Axio Observer A2m Carl Zeiss, Jena, Germany) observation, samples were polished and chemically etched in the solution of 1 g picric acid (North University of China, Taiyuan, China), 14 mL alcohol (North University of China, Taiyuan, China), 2 mL acetic acid (North University of China, Taiyuan, China), and 2 mL distilled water (North University of China, Taiyuan, China). The microstructure and micro-texture of the samples were observed by electron back-scattered diffraction (EBSD, Hitachi SU5000, Tokyo, Japan) at a working voltage of 20 kV, a working distance of 15 mm, and a tilt angle of 70°. Samples for EBSD analysis were initially mechanically polished and then electro-polished with perchloric acid-ethanol reagent (volume ratio 1:9) (North University of China, Taiyuan, China) at −30 °C at a voltage of 15 V for ~120 s. The EBSD data was analyzed by orientation imaging microscopy (OIM) software (EDAX, Philadelphia, PA, USA). The macro-textures of the ED-TD plane of the samples were measured using X-ray diffraction (XRD, Rigaku, Tokyo, Japan).

## 3. Results

**Figure 3:**
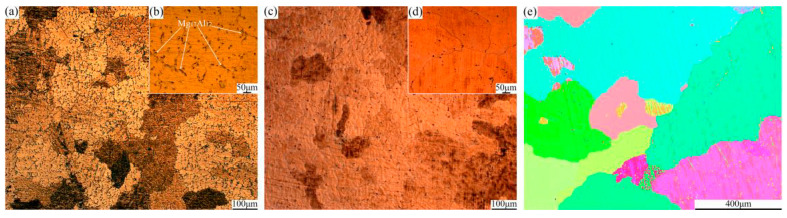
Microstructures of (**a**,**b**) as-cast: (**a**) OM images, (**b**) enlarged in (**a**); and (**c**–**e**) as-homogenized AZ31 alloys: (**c**) OM image, (**d**) enlarged in (**c**), (**e**) IPF image.

**Figure 4:**
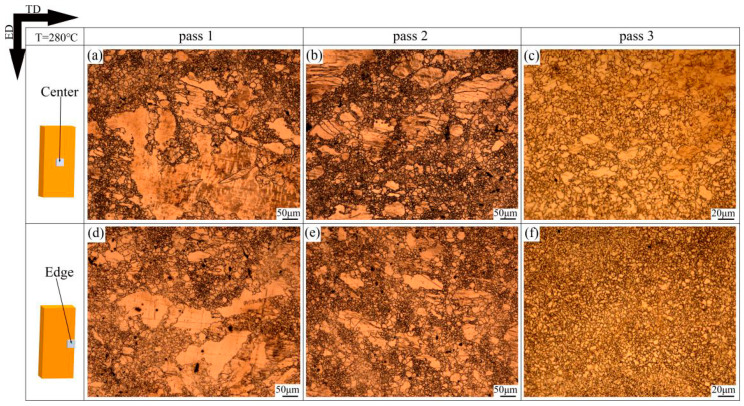
The optical microscope (OM) images of samples after different CEE-AEC passes at 280 °C: (**a**) Center pass 1; (**b**) Center pass 2; (**c**) Center pass 3; (**d**) Edge pass 1; (**e**) Edge pass 2; (**f**) Edge pass 3.

Figure 3 reveals microstructures of as-cast and homogenized samples, and the typical second phase Mg17Al12 can be seen in the as-cast AZ31 sample in Figure 3b. Figure 3c,d demonstrates that the second phase almost disappeared completely in the homogenized AZ31 sample, with an average grain size of 425 μm. The OM images of the center and edge samples after different passes at 280 °C are shown in Figure 4. It is obvious that the microstructures of the deformed samples are effectively refined after each pass of the CEE-AEC deformation. After pass 1, the microstructures are heterogeneous, containing many coarse deformed grains and fine recrystallized grains (Figure 4a,d). As the passes increase, the cumulative strain increases, and the coarse grains are gradually refined. After pass 2, the microstructures of the center and edge samples are almost homogeneous equiaxed grains (Figure 4c,f). In the center region, the average grain sizes decrease from 22.8 μm (pass 1) to 9.8 μm (pass 2). After pass 3, the coarse grains almost disappeared, and the microstructures are fine equiaxed grains.

**Figure 5:**
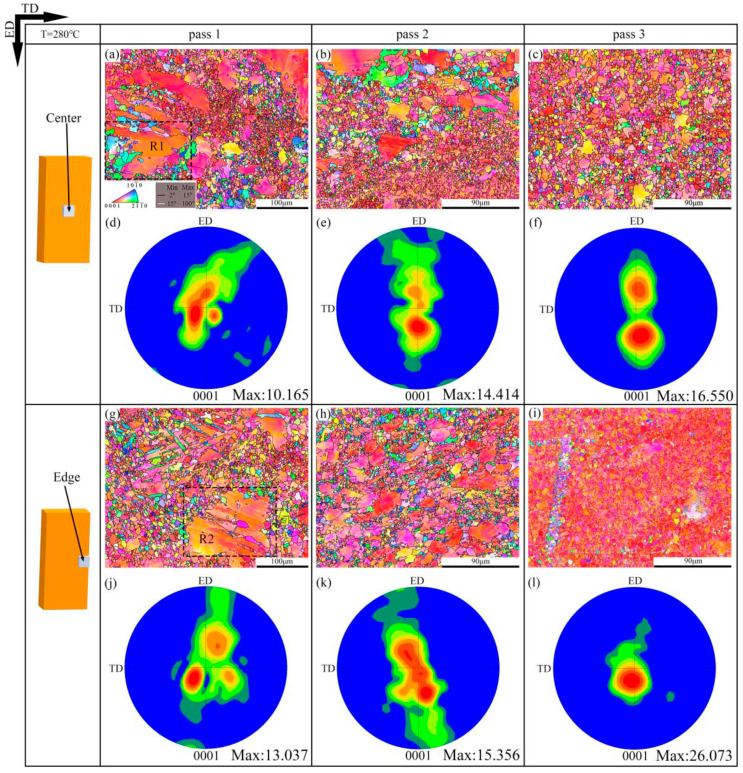
The electron back-scattered diffraction (EBSD) inverse pole figure (ipf) maps and (0001) pole figure maps of the CEE-AEC samples at 280 °C: (**a**) IPF map of Center pass 1; (**b**) IPF map of Center pass 3; (**c**) IPF map of Center pass 3; (**d**) The corresponding (0001) pole figure in (**a**); (**e**) The corresponding (0001) pole figure in (**b**); (**f**) The corresponding (0001) pole figure in (**c**); (**g**) IPF map of Edge pass 1; (**h**) IPF map of Edge pass 2; (**i**) IPF map of Edge pass 3; (**j**) The corresponding (0001) pole figure in (**g**); (**k**) The corresponding (0001) pole figure in (**h**); (**l**) The corresponding (0001) pole figure in (**i**).

**Figure 6:**
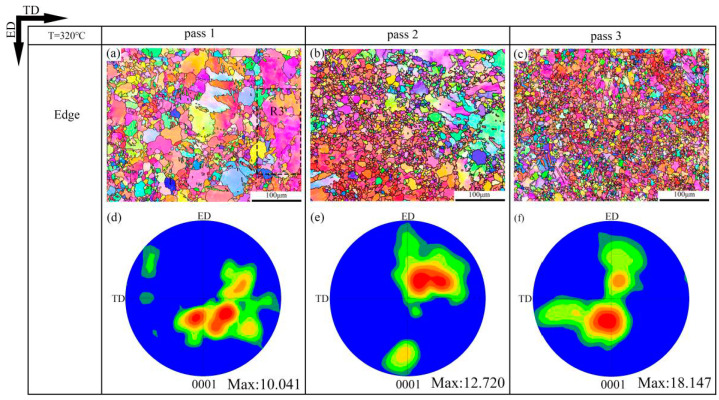
The EBSD inverse pole figure maps and (0001) pole figure maps of the CEE-AEC samples at 320 °C: (**a**) IPF map of Edge pass 1; (**b**) IPF map of Edge pass 2; (**c**) IPF map of Edge pass 3; (**d**) The corresponding (0001) pole figure in (**a**); (**e**) The corresponding (0001) pole figure in (**b**); (**f**) The corresponding (0001) pole figure in (**c**).

**Figure 7:**
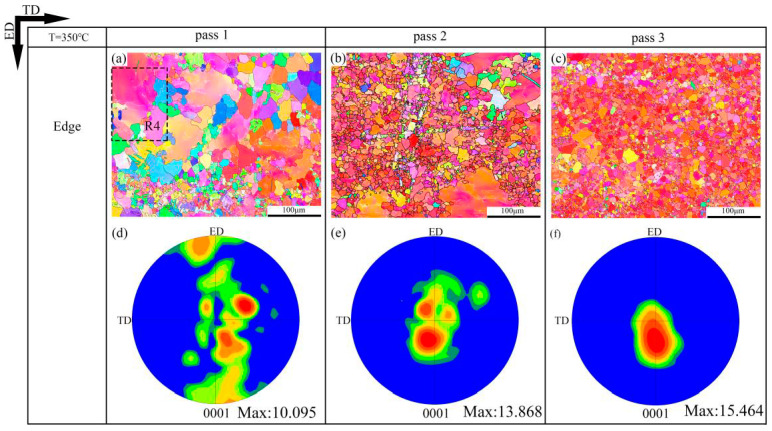
The EBSD inverse pole figure maps and (0001) pole figure maps of the CEE-AEC samples at 350 °C: (**a**) IPF map of Edge pass 1; (**b**) IPF map of Edge pass 2; (**c**) IPF map of Edge pass 3; (**d**) The corresponding (0001) pole figure in (**a**); (**e**) The corresponding (0001) pole figure in (**b**); (**f**) The corresponding (0001) pole figure in (**c**).

**Figure 8:**
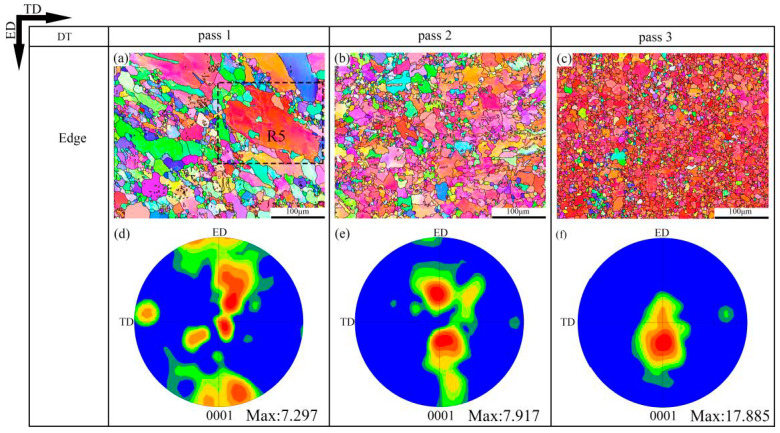
The EBSD inverse pole figure maps and (0001) pole figure maps of the CEE-AEC samples at decreasing temperature: (**a**) IPF map of Edge pass 1; (**b**) IPF map of Edge pass 2; (**c**) IPF map of Edge pass 3; (**d**) The corresponding (0001) pole figure in (**a**); (**e**) The corresponding (0001) pole figure in (**b**); (**f**) The corresponding (0001) pole figure in (**f**).

**Figure 9:**
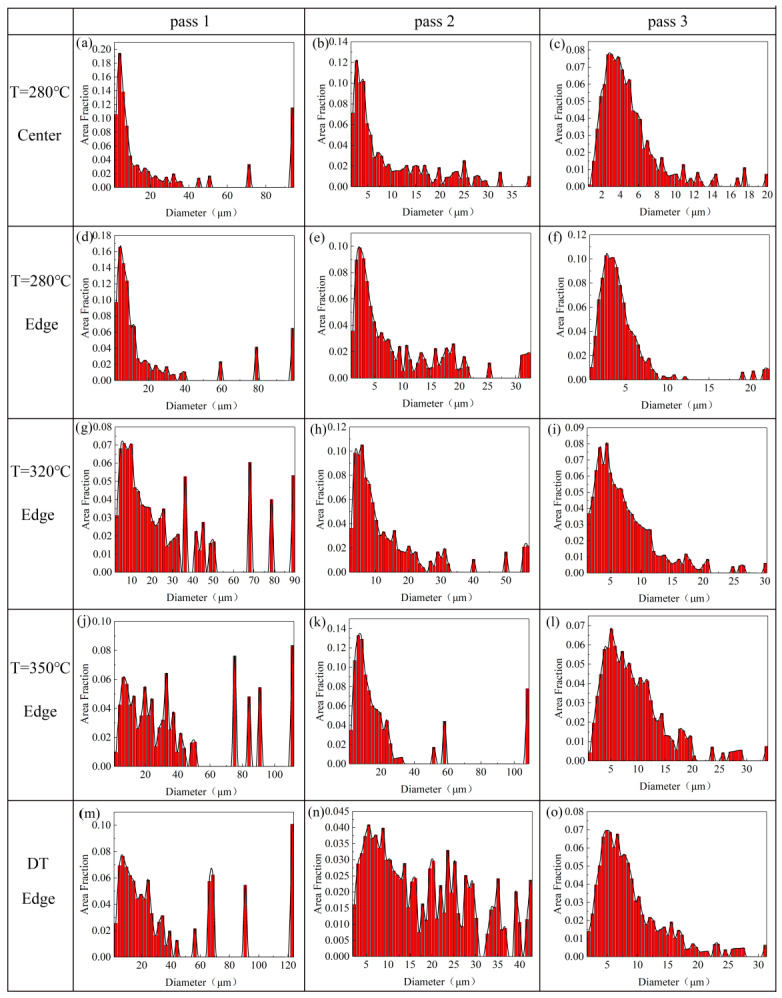
The grain size distribution maps of the CEE-AEC samples at different temperatures: (**a**) Center pass 1 at 280 °C; (**b**) Center pass 2 at 280 °C; (**c**) Center pass 3 at 280 °C; (**d**) Edge pass 1 at 280 °C; (**e**) Edge pass 2 at 280 °C; (**f**) Edge pass 3 at 280 °C; (**g**) Edge pass 1 at 320 °C; (**h**) Edge pass 2 at 320 °C; (**i**) Edge pass 3 at 320 °C; (**j**) Edge pass 1 at 350 °C; (**k**) Edge pass 2 at 350 °C; (**l**) Edge pass 3 at 350 °C; (**m**) Edge pass 1 at DT; (**n**) Edge pass 2 at DT; (**o**) Edge pass 3 at DT.

The low angle grain boundaries (LAGBs, misorientation angles between 2° and 15°) marked with white lines and the high angle grain boundaries (HAGBs, misorientation angles higher than 15°) shown by black lines are demonstrated in Figure 5, Figure 6, Figure 7 and Figure 8. The EBSD inverse pole figure maps and (0001) pole figure maps of the center and edge samples at 280 °C are illustrated in Figure 5. After one pass, the microstructures of the center and edge samples exhibit a typical bimodal distribution, which consists of coarse grains of 40–100 μm and fine grains of <10 μm [27]. From Figure 9a,d, it is also obvious that the microstructures of AZ31 alloys after one pass are heterogeneous. After three passes, the microstructures of the center and edge samples are sharply refined, and their average grain sizes are 5.1 μm and 4.5 μm, respectively. It can also be seen from Figure 9c,f that the microstructure of the edge sample is more uniform than that of the center sample, and the percentage of grains with a size of <10 μm is larger. This is consistent with the finite element simulation results where the effective strain at the edge is larger than the center [25]. In addition, the basal textures of the center and edge samples are shown in Figure 5d–f,j–l, respectively. The maximum intensities of the basal textures of the center and edge samples after one pass are 10.165 and 13.037, respectively. As the passes increase, the grains orientation tends to be consistent, and the maximum intensities of the basal textures gradually increase. The maximum intensity of the basal texture of the 280 °C-edge samples is 26.073, and it tilted by approximately ±25° from ND to ED. In addition, Figure 5f shows that a bimodal basal texture is formed in the ED × TD plane of the 280 °C-center sample with a maximum intensity of 16.550 and a slope of about ±40° from ND to ED. This inclined bimodal basal texture has also been reported in past studies and proved that it can significantly improve the mechanical properties of AZ31 alloy [28,29,30]. Han et al. [31] investigated that the AZ31 alloy prepared by accumulated extrusion bonding also formed a bimodal basal texture along the ED, which was caused by the initiation of pyramidal <c + a> slip during the extrusion [32].

The EBSD inverse pole figure maps and (0001) pole figure maps of the 320 °C-edge, 350 °C-edge and DT samples are shown in Figure 6, Figure 7 and Figure 8, respectively. The grain size distribution maps of the CEE-AEC samples at different temperatures are demonstrated in Figure 9. Table 1 lists the average grain sizes of samples at different temperatures. Obviously, microstructures of all samples show a typical bimodal distribution after one pass (Figure 9g,j,m), and the average grain sizes of samples decrease as the passes increase. After three passes, the average grain size of the 350 °C-edge sample is 9.3 μm, which is the largest of all samples. This indicates that the temperature significantly affects the degree of grain refinement in the deformed samples. The reason is that the higher deformation temperature promotes the DRX nucleation but also accelerates the growth of the grains so that the newly formed fine equiaxed grains absorb energy and grow up quickly during the deformation process [33]. In addition, the basal textures of different samples also deflected, and the maximum intensities gradually increased with the passes increase. The microstructures are basically replaced by DRX grains after three passes of CEE-AEC deformation. Due to the shear strain caused by the asymmetrical cavity, these fine grains have the same tendency to rotate during the deformation process, thus causing the maximum intensities of the basal textures to rise. After three passes, the maximum intensity of the basal texture of the 320 °C-edge sample 320 °C is 18.147, which is deflected from the ND to the ED by approximately ±35°. The maximum intensity of the basal texture of the 350 °C-edge sample is 15.464, which is deflected from ND to ED by approximately ±30°. The maximum intensity of the basal texture of the DT-edge sample is 17.885, and the deflection from ND to ED is about ±30°.

**Figure 10:**
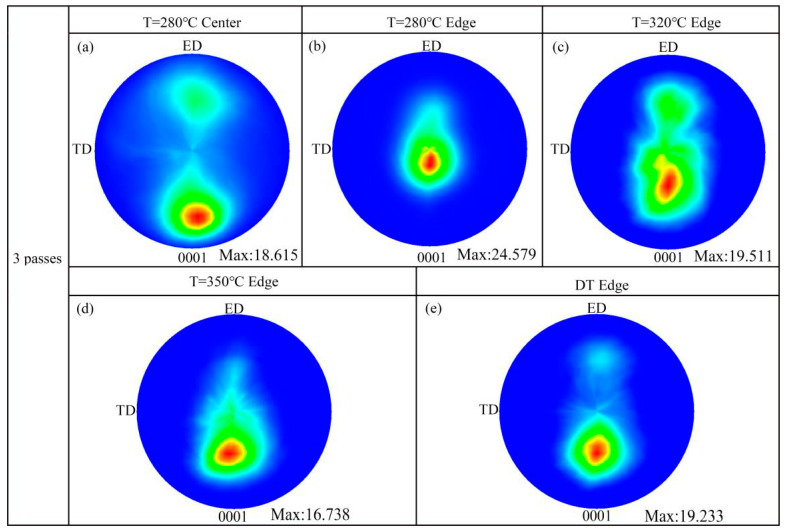
The (0001) macro-textures of different samples: (**a**) Center pass 3 at 280 °C; (**b**) Edge pass 3 at 280 °C; (**c**) Edge pass 3 at 320 °C; (**d**) Edge pass 3 at 350 °C; (**e**) Edge pass 3 at 320 °C.

The (0001) macro-textures of different samples after pass 3 are shown in Figure 10. The maximum intensities and deflection angles of the micro-textures (Figure 5, Figure 6, Figure 7 and Figure 8) are almost consistent with the macro-textures (Figure 10). The maximum intensity of the basal texture of the 280 °C-edge sample is 24.579, which is the highest among all samples. In addition, Figure 10c shows that the bimodal texture is also generated in the 320 °C-edge sample. Furthermore, the (0001) pole figure in the 280 °C-center sample shows a completely split basal texture, which is defined as Zn-texture [29]. The deflection orientation of the basal textures is conducive to the basal slip of the Mg alloys, which improves the room-temperature formability of theAZ31 alloy [34,35,36].

**Figure 11:**
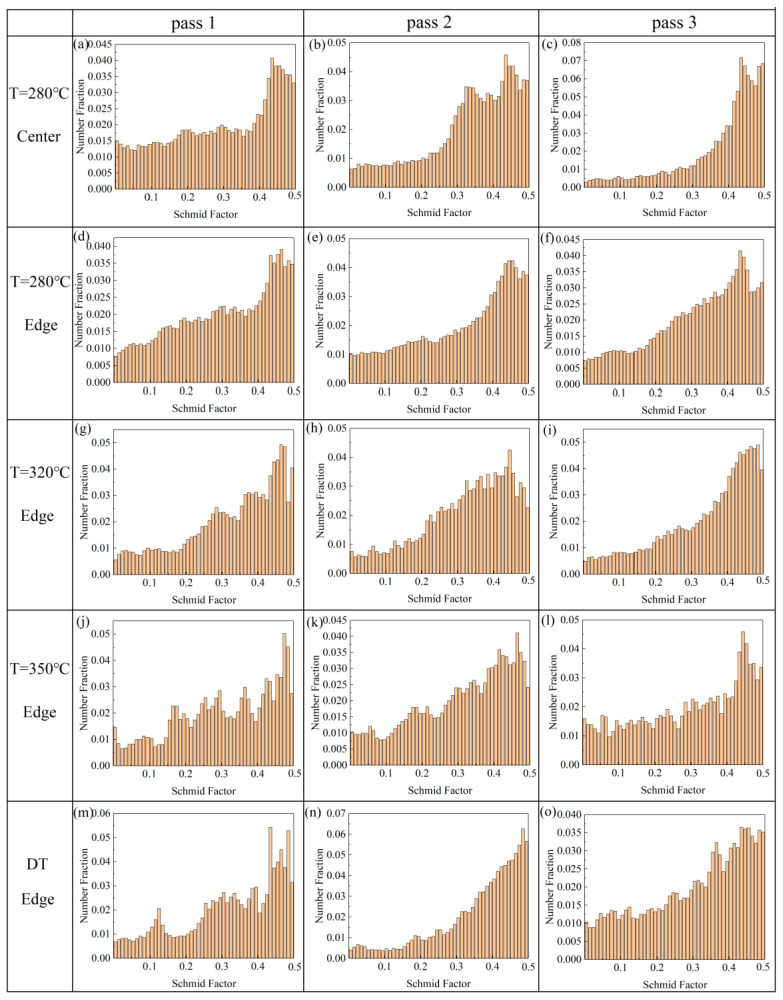
The (0001) <11 − 20> Schmid factor distribution maps of different samples: (**a**) Center pass 1 at 2800 °C; (**b**) Center pass 2 at 280 °C; (**c**) Center pass 3 at 280 °C; (**d**) Edge pass 1 at 280 °C; (**e**) Edge pass 2 at 280 °C; (**f**) Edge pass 3 at 280 °C; (**g**) Edge pass 1 at 320 °C; (**h**) Edge pass 2 at 280 °C; (**i**) Edge pass 3 at 320 °C; (**j**) Edge pass 1 at 350 °C; (**k**) Edge pass 2 at 350 °C; (**l**) Edge pass 3 at 350 °C; (**m**) Edge pass 1 at DT; (**n**) Edge pass 2 at DT; (**o**) Edge pass 3 at DT.

The (0001) <11 − 20> Schmid factor distribution maps of different samples are shown in Figure 11. Table 2 lists the average values of (0001) <11 − 20> Schmid factor of different samples. In Figure 11, after one pass, the Schmid factor distribution maps of all samples have a peak value over 0.4, and the average values of the Schmid factor are higher than 0.3. As the passes increase, the average values of the Schmid factors of the 280 °C-center sample and 320 °C-edge sample increase, and they reach 0.38 and 0.36 after three passes, respectively. Compared to one pass, the percentage of the Schmid factors over 0.3 increases, although the average values of other samples did not increase significantly after three passes. It is well known that basal slip is the predominant mode in deformation of Mg alloys at room temperature [37,38]. In the same situation, a higher average value of Schmid factor determines that the basal slip requires a lower critical resolved shear stress (CRSS); that is, a higher Schmid factor is easier to dislocation movement on the (0001) basal plane. Furthermore, the Schmid factor with an average value >0.3 indicates a favorable, soft orientation for basal slip along ED, which is beneficial to improve the ductility of the AZ31 alloy in the ED. At present, the main purpose of texture control is to weaken the texture with deflection to improve the ductility of AZ31 alloys, but weakening the texture will deteriorate the yield strength [31,39]. In this experiment, the basal textures of the samples prepared by the CEE-AEC process were deflected while the maximum intensities were increased, which resulted in improved ductility without reducing the strength. In general, the AZ31 alloy prepared by the CEE-AEC process has a strong inclined texture, and the inclined texture in the ED is beneficial to form a soft orientation that is easy to dislocation slip, thereby reducing the CRSS of the basal slip.

**Figure 12:**
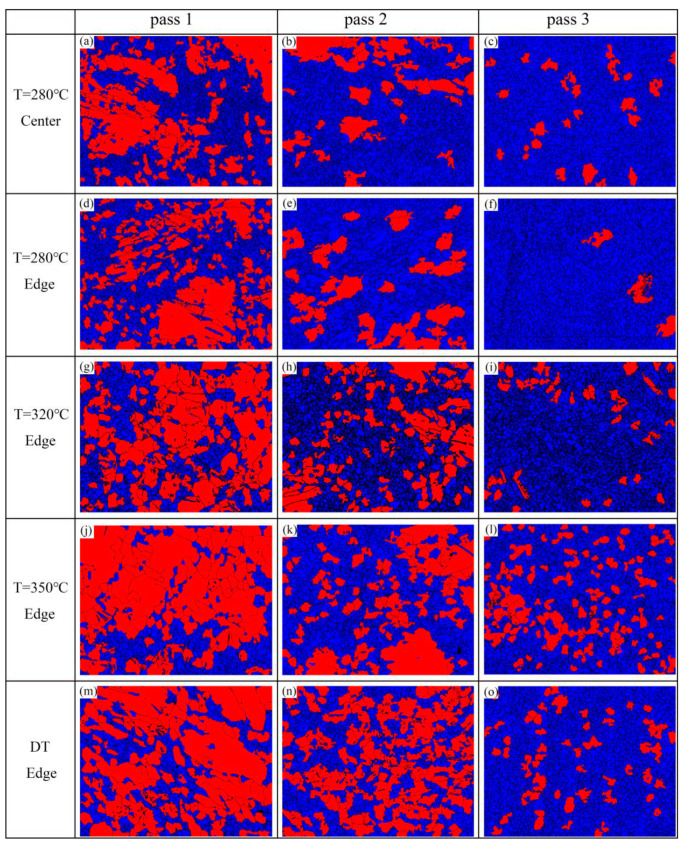
The EBSD maps distinguished recrystallization region (blue area) and deformed region (red area) of different samples: (**a**) Center pass 1 at 280 °C; (**b**) Center pass 2 at 280 °C; (**c**) Center pass 3 at 280 °C; (**d**) Edge pass 1 at 280 °C; (**e**) Edge pass 2 at 280 °C; (**f**) Edge pass 3 at 280 °C; (**g**) Edge pass 1 at 320 °C; (**h**) Edge pass 2 at 320 °C; (**i**) Edge pass 3 at 320 °C; (**j**) Edge pass 1 at 350 °C; (**k**) Edge pass 2 at 350 °C; (**l**) Edge pass 3 at 350 °C; (**m**) Edge pass 1 at DT; (**n**) Edge pass 2 at DT; (**o**) Edge pass 3 at DT.

In order to compare the DRX behavior between different samples, the EBSD distribution maps of DRXed grains (marked as blue color) and deformed grains (marked as red color) are shown in Figure 12. The area fractions of DRXed grains of different samples are summarized in Table 3. The area fraction of DRXed grains of the 280-edge sample increased from 55.7% (one pass) to 96.8% (three passes); almost complete dynamic recrystallization occurred, which is the highest among all samples. The DRX mechanism of different samples during CEE-AEC deformation will be further analyzed.

**Figure 13:**
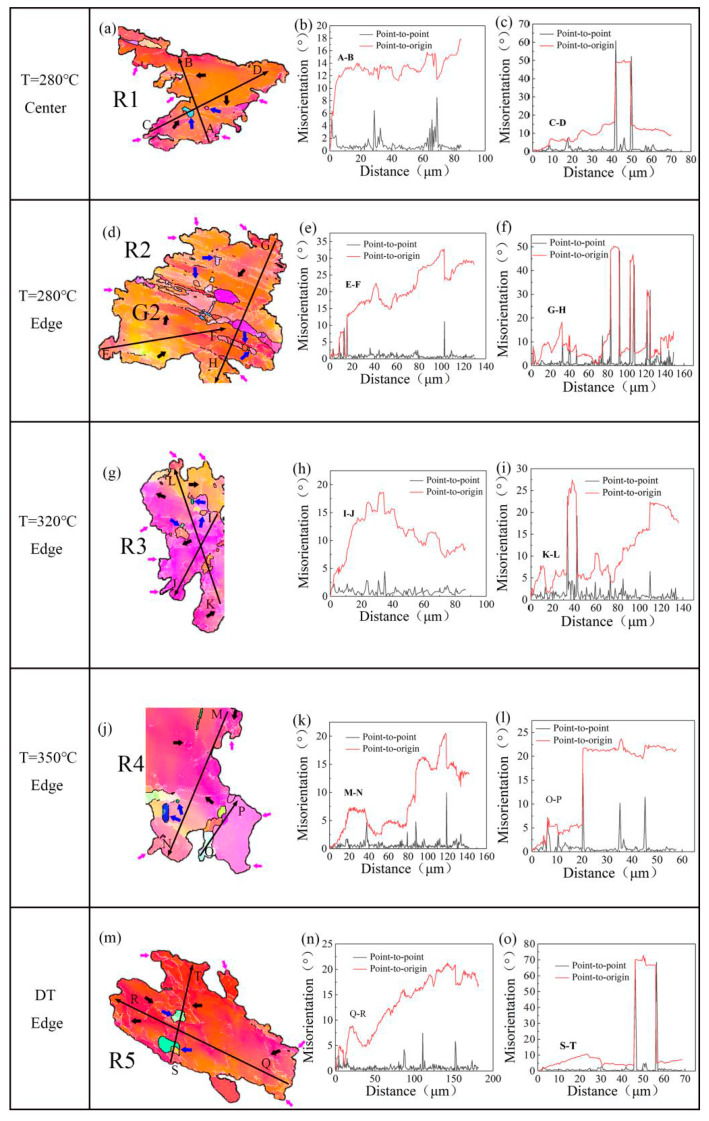
The DRX behavior of original grains in different samples after pass 1 is shown in the first column of the images, and the inverse pole figure maps the line profiles of the misorientation angle along the arrows in the corresponding inverse pole figure maps as shown in the central and right columns: (**a**) Center pass 1 at 280 °C; (**b**) Line profiles of arrow AB; (**c**) Line profiles of arrow CD; (**d**) Edge pass 1 at 280 °C; (**e**) Line profiles of arrow EF; (**f**) Line profiles of arrow GH; (**g**) Edge pass 1 at 320 °C; (**h**) Line profiles of arrow IJ; (**i**) Line profiles of arrow KL; (**j**) Edge pass 1 at 350 °C; (**k**) Line profiles of arrow MN; (**l**) Line profiles of arrow OP; (**m**) Edge pass 1 at DT; (**n**) Line profiles of arrow QR; (**o**) Line profiles of arrow ST.

The grains selected from the different samples after one pass (highlighted by dashed boxes in Figure 5, Figure 6, Figure 7 and Figure 8) are shown in Figure 13. Figure 13d reveals the DRX behavior of the deformed grains in the region R2 of the 280 °C-edge sample (highlighted with a dotted box in Figure 5g), and the point-to-point misorientation angles, and the point-to-origin misorientation angles of the arrows EF and GH shown in Figure 13e,f, respectively. It is clear in Figure 13d that the coarse grain G2 is composed of red- and orange-color regions. The line profile of point-to-origin along the arrow EF (Figure 13e) shows that the misorientation angle gradually increases to 33°, which indicates that a continuous orientation change has occurred in G2. In addition, lots of LAGBs in the G2, as indicated by the black arrows, which is usually caused by the accumulation of dislocations or the formation of sub-grains in the deformed grains [28]. As the cumulative strain increases, these LAGBs can be transformed into HAGBs by trapping more moving dislocations. Eventually, new DRXed grains (marked as blue arrows) are obtained [40,41] and expressed as a sudden rise in the point-to-point curve in Figure 13f. This is a typical continuous dynamic recrystallization (CDRX) mechanism [28,42]. In addition, it can be observed from Figure 13d that the grain boundaries of the coarse grains G2 are serrated (marked as purple arrows), and the sub-grains are separated from the coarse grains through the sub-grain boundaries, and new DRXed grains are formed along the serrated grain boundaries, which is a typical discontinuous dynamic recrystallization (DDRX) mechanism [43]. Analysis of other regions in Figure 13 shows that CDRX and DDRX also occurred in other samples prepared by CEE-AEC process.

## 4. Conclusions

In this paper, the evolution process of the microstructure and texture of the AZ31 alloy sample prepared by the asymmetric extrusion cavity cyclic expansion-extrusion at different temperatures was studied. The results are summarized as follows:

(1) During CEE-AEC processing, the AZ31 alloy exhibits a typical bimodal microstructure, with coarse deformed grains surrounded by fine DRXed grains. As the passes increase, the coarse grains of all samples are almost replaced by fine equiaxed grains. When the deformation temperature is 280 °C, the average grain size in the edge region is smaller than that in the center region. In addition, with deformation temperature increases, the degree of grain refinement of the AZ31 alloy samples in the edge regions decreases; the average grain size of the 280 °C-edge sample was 4.5 μm (pass 3), which is the smallest of all samples.

(2) Due to the presence of asymmetrical cavities in the CEE-AEC dies, the basal textures of the samples were deflected after deformation, which forms a soft orientation that is easy to basal slip. As the deformation passes increase, the basal texture intensities of all samples increase. Moreover, unusual bimodal textures are formed in the 280 °C-center sample and the 320 °C-edge sample, and the tilt angle of basal texture of the 280 °C-center sample from ND to ED is about ±40°, which is the largest of all samples.

(3) The CDRX and DDRX that occurred during the CEE-AEC deformation process were the main mechanisms for the grain refinement of the AZ31 alloy.

## Figures and Tables

**Table 1:** The average grain sizes of the CEE-AEC samples at different temperatures.

Samples	Average Grain Size (μm)		
	Pass 1	Pass 2	Pass 3
T = 280 °C Center	22.8	9.8	5.1
T = 280 °C Edge	20.4	9.0	4.5
T = 320 °C Edge	24.5	13.8	7.3
T = 350 °C Edge	40.3	22.1	9.3
DT Edge	39.8	17.8	8.9

**Table 2:** Average values of (0001) <11 − 20> Schmid factor of different samples.

Samples	Pass 1	Pass 2	Pass 3
T = 280 °C Center	0.3	0.34	0.38
T = 280 °C Edge	0.31	0.32	0.31
T = 320 °C Edge	0.33	0.33	0.36
T = 350 °C Edge	0.31	0.31	0.3
DT Edge	0.33	0.37	0.31

**Table 3:** The area fraction of dynamic recrystallization (DRX)ed grains of different samples.

Samples	DRX Fraction (%)		
	Pass 1	Pass 2	Pass 3
T = 280 °C Center	54.6	75.6	92.1
T = 280 °C Edge	55.7	77.4	96.8
T = 320 °C Edge	41.8	66.6	90.6
T = 350 °C Edge	30.5	42.7	82.5
DT Edge	33.8	48.3	86.5
